# Novel Neurostimulation of Autonomic Pelvic Nerves Overcomes Bladder-Sphincter Dyssynergia

**DOI:** 10.3389/fnins.2018.00186

**Published:** 2018-03-21

**Authors:** Wendy Yen Xian Peh, Roshini Mogan, Xin Yuan Thow, Soo Min Chua, Astrid Rusly, Nitish V. Thakor, Shih-Cheng Yen

**Affiliations:** ^1^Singapore Institute for Neurotechnology, National University of Singapore, Singapore, Singapore; ^2^Department of Biomedical Engineering, National University of Singapore, Singapore, Singapore; ^3^Department of Electrical and Computer Engineering, National University of Singapore, Singapore, Singapore; ^4^Biomedical Engineering, School of Medicine, Johns Hopkins University, Baltimore, MD, United States

**Keywords:** high frequency stimulation, neurostimulation, bladder, pelvic nerves, dyssynergia, micturition

## Abstract

The disruption of coordination between smooth muscle contraction in the bladder and the relaxation of the external urethral sphincter (EUS) striated muscle is a common issue in dysfunctional bladders. It is a significant challenge to overcome for neuromodulation approaches to restore bladder control. Bladder-sphincter dyssynergia leads to undesirably high bladder pressures, and poor voiding outcomes, which can pose life-threatening secondary complications. Mixed pelvic nerves are potential peripheral targets for stimulation to treat dysfunctional bladders, but typical electrical stimulation of pelvic nerves activates both the parasympathetic efferent pathway to excite the bladder, as well as the sensory afferent pathway that causes unwanted sphincter contractions. Thus, a novel pelvic nerve stimulation paradigm is required. In anesthetized female rats, we combined a low frequency (10 Hz) stimulation to evoke bladder contraction, and a more proximal 20 kHz stimulation of the pelvic nerve to block afferent activation, in order to produce micturition with reduced bladder-sphincter dyssynergia. Increasing the phase width of low frequency stimulation from 150 to 300 μs alone was able to improve voiding outcome significantly. However, low frequency stimulation of pelvic nerves alone evoked short latency (19.9–20.5 ms) dyssynergic EUS responses, which were abolished with a non-reversible proximal central pelvic nerve cut. We demonstrated that a proximal 20 kHz stimulation of pelvic nerves generated brief onset effects at lower current amplitudes, and was able to either partially or fully block the short latency EUS responses depending on the ratio of the blocking to stimulation current. Our results indicate that ratios >10 increased the efficacy of blocking EUS contractions. Importantly, we also demonstrated for the first time that this combined low and high frequency stimulation approach produced graded control of the bladder, while reversibly blocking afferent signals that elicited dyssynergic EUS contractions, thus improving voiding by 40.5 ± 12.3%. Our findings support advancing pelvic nerves as a suitable neuromodulation target for treating bladder dysfunction, and demonstrate the feasibility of an alternative method to non-reversible nerve transection and sub-optimal intermittent stimulation methods to reduce dyssynergia.

## Introduction

Recent developments in the field of bioelectronic medicine call for new innovations in stimulating the nervous system for treating diseases (Kristoffer Famm, 2013; Birmingham et al., 2014; Reardon, 2014), including the targeting of peripheral nerves that modulate the function of visceral organs, such as the urinary bladder (de Groat and Tai, 2015; Lee et al., 2015; McGee et al., 2015) and spleen (Pavlov and Tracey, 2012). Dysfunction of the urinary bladder can stem from neurological impairment (Yoshimura and Chancellor, 2003), such as spinal cord injuries (Taweel and Seyam, 2015), and can affect the process of micturition, which is comprised of a coordinated sequence of contraction of bladder detrusor muscles, and relaxation of sphincter muscles to expel urine stored in the bladder through the urethra. This process requires descending inputs from the pontine micturition center to excite sacral parasympathetic neurons, which then synapse onto postganglionic neurons in the pelvic plexus in humans and the major pelvic ganglion (MPG) in rodents, to contract the smooth detrusor muscles. The descending inputs also inhibit cholinergic sacral motor neurons whose axons travel within the pudendal nerve that excites striated external urethral sphincter (EUS) muscles (Fowler et al., 2008). Implantation of a neuromodulation device is a possible alternative when neither non-surgical bladder management options (such as intermittent catheterization, which may not be suitable for patients who are physically unable to perform the procedure themselves, or have psychological barriers to performing the procedure) nor pharmacological agents (such as botulinum toxin to reduce either detrusor overactivity or sphincter activity) are able to achieve the desired clinical results (Gaunt and Prochazka, 2005; Dorsher and McIntosh, 2012). Neurostimulation methods are also more reversible than pharmacological methods, and have the potential to restore voluntary control of micturition. Previous electrical neuromodulation techniques for restoring bladder control have typically focused on stimulating anatomically larger or more accessible neural targets, such as the sacral nerve roots (Hohenfellner et al., 2001a; Possover et al., 2010; Su et al., 2012; Bruns et al., 2015; Matzel et al., 2017), the spinal cord (Gad et al., 2014; Guiho et al., 2017; Pettigrew et al., 2017), or the pudendal nerve (Boggs et al., 2006; Gaunt and Prochazka, 2009; Chen et al., 2013; McGee and Grill, 2014; Yang et al., 2014). However, modulating the activity of these nerve targets to mimic natural micturition is not without challenges. For example, voiding of urine in spinal cord injured patients can be triggered by sacral anterior root stimulation (SARS) using the FDA-approved Fintech Brindley system (Brindley, 1994), also known as the VOCARE Bladder system in the United States (although it is currently unavailable there, McGee et al., 2015). However, SARS requires a permanent rhizotomy of the sensory dorsal roots to prevent bladder or detrusor sphincter dyssynergia, which involves undesirable EUS muscle contractions that limit voiding (Kirkham et al., 2002; Martens and Heesakkers, 2011). An alternative approach is to appropriately stimulate the afferent fibers of the pudendal nerve that convey sensory signals from the urethra, or its associated branches. This approach has been shown to elicit voiding, but its success is dependent on intact spinal reflexes (Boggs et al., 2005; Woock et al., 2008; Yoo et al., 2008), and has limited voiding efficacy (Langdale and Grill, 2016).

In contrast to these more commonly stimulated nerve locations, the smaller, distal pelvic nerves branches, which carry efferent parasympathetic inputs to contract the detrusor muscles of the bladder wall (Langworthy, 1965; Purinton et al., 1973), remain under-explored as a neuromodulation target for restoring voluntary control of the emptying of bladder (de Groat and Tai, 2015; McGee et al., 2015). The advantage of manipulating the pelvic nerve is that it could provide a more direct and specific control of bladder contractions than central nerve targets or afferent fibers. However, the pelvic nerve is a mixed nerve, containing not only parasympathetic efferent inputs to the bladder, but also sensory afferent fibers (including Aδ and C fibers) that convey bladder sensation from mechanoreceptor and chemoreceptor nerve endings in the bladder wall toward the spinal cord in rats (Purinton et al., 1973; Sengupta and Gebhart, 1994; Shea et al., 2000). Histological studies in rats have revealed that the pelvic nerve is made up of a mixture of myelinated and unmyelinated fibers, with the majority of the large myelinated fibers being sensory as they were eliminated after spinal ganglionectomy (Purinton et al., 1973). Unlike rodent models, in which there is a MPG on each side with distinct pelvic nerve branches adjoining the ganglion, the human pelvic splanchnic nerves emerge from the sacral roots S2-4, and contain parasympathetic efferents that merge into the inferior hypogastric plexus or pelvic plexus, which contains many ganglion with interconnected nerve branches (Wozniak and Skowronska, 1967; Dail, 1996; de Groat et al., 2015). Characterization of the nerve fibers in the pelvic plexus in humans were mostly limited to cadavers, and based on immunohistochemical markers (such as vasoactive intestinal peptides) that demonstrate a mixture of parasympathetic, sympathetic, and sensory fibers (Hinata et al., 2014; Jang et al., 2015). Electrical stimulation of the pelvic nerve has previously been performed to investigate its functional effects on the urinary bladder and urethra in rodents (Chang, 2006; Crook and Lovick, 2017), dogs (Creed and Tulloch, 1978; Andersson et al., 1990), pigs (Dalmose et al., 2002). In humans, the same stimulation had varying success, but also revealed difficult issues such as pelvic nerve scarring due to chronic stimulation, and difficulty with electrode placement (Hald, 1969; Burghele, 1973; Possover et al., 2007). Overall, many of these studies revealed that electrical stimulation of the pelvic nerve can evoke intravesical pressure increases. However, sphincter contractions have also been observed during pelvic nerve stimulation (Holmquist and Olin, 1968; Burghele, 1973; Chang, 2006; Crook and Lovick, 2017). This stimulation-evoked bladder-sphincter dyssynergia is likely due to the artificial activation of the pelvic-to-pudendal nerve pathway, also known as the guarding reflex (Fowler et al., 2008; McGee et al., 2015), causing co-activation of the EUS muscle contraction during bladder wall contraction. Thus, a novel strategy for electrical neuromodulation of pelvic nerve, specifically the activation of its efferent pathway, is required for more effective bladder emptying.

Manipulating the activity of distinct axonal populations that have no clear spatial anatomical organization within the mixed nerves remains technically challenging in clinical settings, although promising progress has been made recently with genetic targeting techniques (Llewellyn et al., 2010; Chang et al., 2015). The technique of kilohertz frequency electrical stimulation (KHFS) has emerged over the years as an effective method of blocking nerve conduction at the site of electrode placement (Bhadra and Kilgore, 2005; Kilgore and Bhadra, 2014; Patel and Butera, 2015). Recently, KHFS has also been used to achieve more selective activation of the efferent pathway within the vagus nerve (Patel et al., 2017). Notably, KHFS of the ventral sacral roots has been shown to block micturition (Chew et al., 2013), while KHFS of the pudendal nerve to block nerve conduction of the motor branches (Tai et al., 2004; Bhadra et al., 2006; Yang et al., 2014) has been shown to prevent untimely EUS contractions. Although KHFS had been applied to pelvic nerve branches to prevent incontinence (Crook and Lovick, 2017), its efficacy of using KHFS to block activation of the afferent pathway during pelvic nerve stimulation in order to reduce bladder-sphincter dyssynergia is unclear.

In this study, we determined if a novel approach of electrical neuromodulation of distal pelvic nerve branches could evoke micturition without bladder-sphincter dyssynergia. We combined distal low frequency stimulation and proximal KHFS unilaterally along the pelvic nerves in anesthetized female rats, and monitored intravesical pressure, EUS muscle activity, and voiding outcomes. Our results showed that this new approach of stimulating pelvic nerves improved urine output, reduced unwanted EUS contractions, and decreased voiding-related bladder pressure, consistent with reduced bladder-sphincter dyssynergia. We conclude this paper with a discussion on advancing neuromodulation of pelvic nerves as a future therapeutic option for bladder dysfunction.

## Methods

### Animal subjects

Adult female Sprague-Dawley rats (200–320 g) were used in this study (*N* = 41 rats). The animals were housed in pairs in individually ventilated cages, maintained in a 22–24°C room with a 12 h light–dark cycle, and given *ad libitum* access to food and water. The animals were acclimatized to the housing conditions for at least 1 week prior to experiments. All procedures were performed in accordance with protocols approved by the Institutional Animal Care and Use Committee of the National University of Singapore. Female rats were used as the pelvic nerves lateral and central to the MPG were easily accessible for combined low and high frequency stimulation. In addition, female rats had been commonly used exclusively in studies involving bladder neuromodulation (Peng et al., 2008; Su et al., 2012; Shi et al., 2013; Langdale and Grill, 2016).

### Surgery

Each rat was anesthetized with a mixture (0.2 ml/100 g) of ketamine (37.5 mg/ml) and xylazine (5 mg/ml) intraperitoneally (I.P.) for induction, and a supplementary dose of 0.1 ml/100 g was injected I.P. for maintenance as required. The animal was placed in the supine position, and kept warm with a water-circulating heating pad (40°C) during the experiment. Using aseptic techniques, a ventral midline incision of the lower abdomen was made to expose the bladder. In order to measure intravesical pressure, a small cut was made in the top of the bladder dome, and a catheter (C30PU-RCA1302, Instech Laboratories Inc., PA, USA) was inserted into the bladder and secured with a 4-0 silk suture. In order to expose the pelvic nerve (~2 mm exposed nerve length) for electrical stimulation, the midline incision was extended laterally, and the underlying muscles were cut. Adipose tissues and connective tissue were gently teased apart or removed to expose the pelvic nerve branches. In order to record muscle activity from the EUS in a subset of experiments, the pubic bone overlying the urethra was cut and fat tissues were teased apart.

### Pressure measurement and analysis

The saline-filled bladder catheter (C30PU-RCA1302, Instech Laboratories Inc, PA, USA) was connected to a pressure sensor (Transpac® IV, ICU Medical Inc., CA, USA) and an infusion pump (KD Scientific, MA, USA) via 3-way stopcock. Saline was infused into the bladder at 40 μl/min when necessary to refill the bladder after voiding, and to keep pressures consistent during repeated stimulation trials, as well as between intact nerve and proximally transected nerve conditions. During interleaved control trials (low frequency stimulation only) and blocking trials (combined low and high frequency stimulation), saline was infused into the bladder to replace previously voided output, and baseline pressure levels were visually monitored. Control and block trials were interleaved to test for reversibility of KHFS, and to minimize any cumulative effects of KHFS overtly affecting one condition over another. All pressure data were amplified using a customized pressure amplifier, and then sampled at 20 kHz using either a PicoScope® 4424 acquisition board (Pico Technology, UK) or Intan RHD 2000 system (Intan Technologies, CA, USA). All acquired data were then analyzed using customized MATLAB software (MathWorks, MA, USA). After data sampling, pressure data was then low-pass filtered at 30 Hz, and a 5 s period prior to each electrical stimulation epoch was used to define the baseline to calculate the change in intravesical pressures during stimulation. Peak pressure was identified as the maximum increase in pressure during the stimulation period. Post-stimulation change in pressure was calculated as the maximum decrease in pressure within a 20-s long window after the end of stimulation epoch. Area under the curve (AUC) of the pressure signal during the stimulation period was calculated to quantify the overall pressure profile.

### Nerve stimulation

Hook electrodes made from platinum iridium wires (A-M systems, 0.005″ bare, 0.008″ coated) were implanted onto the pelvic nerve branches unilaterally, and were used for both low and high frequency electrical stimulation. The distinct nerve branches were not teased apart, but were kept together to be contacted by the same pair of electrodes. Distances between hook electrode leads varied between 250 and 1,000 μm. During combined low and kilohertz frequency stimulation (KHFS), the pair of hook electrodes was separated between 500 and 1,000 μm. Silicone elastomer (Kwik-Sil, World Precision Instruments, FL, USA) was used to encase the electrode-nerve interface, if necessary. A commercial isolated stimulator (A-M systems model 2100, WA, USA) was used to deliver low frequency (10 or 20 Hz) biphasic rectangular pulses for pelvic nerve stimulation. The duration of the low frequency stimulation was 5 s, unless indicated otherwise. The phase widths of the biphasic pulses were either 150 or 300 μs, with no interphase delay. The A-M systems Model 4100 stimulator was used to deliver KHFS (20 kHz, 25 μs phase width, no interphase delay) comprising of biphasic rectangular pulses during blocking experiments. KHFS was set at 20 kHz after performing some preliminary testing at 10, 20, and 40 kHz. Low frequency stimulation amplitudes ranged from 25 to 500 μA, while KHFS blocking amplitudes ranged from 100 to 1,000 μA. In order to enable data synchronization, stimulation pulse markers were sent from the stimulators to data acquisition boards collecting pressure and electromyography (EMG) data simultaneously.

### Pelvic nerve transections

In a subset of experiments where unilateral pelvic nerves were transected, the central sections of the nerve branches proximal to the stimulation electrodes were further exposed and transected using surgical vanna scissors. In order to compare the stimulation effects of intact vs. unilateral proximal transected pelvic nerve, three consecutive trials of 10 Hz stimulation (300 μs phase width, 400 μA) were first performed on the intact nerve, and then followed by identical stimulation of the transected nerve.

### Urine detection and volume measurement

For temporal detection of voiding events (either stimulation-evoked or spontaneous), a voltage divider circuit with a pair of open wires was placed immediately outside the urethral meatus but not in direct contact with the animal. Urine outflow connecting the pair of wires allowed increased positive voltages to be acquired by the same data acquisition board collecting pressure, stimulation, or EMG data. Urine was collected via Eppendorf tubes placed outside the meatus, and later quantified using a precision weighing scale. The measured weight was then converted to volume using an average density measured from multiple samples.

### External urethral sphincter (EUS) electromyography (EMG)

In order to record EMG from the EUS, a pair of fine stainless steel wires (304, California Fine Wire, CA, USA) with exposed tips was inserted on top of the exposed urethra beneath the dissected pubic bone. Before the start of the stimulation experiments, the quality of the EMG recordings was first verified by inducing bladder-filling evoked voiding responses to ensure that EUS EMG recordings associated with voiding were obtained. EMG signals were amplified by using an Intan preamplifier 2216, and acquired at 20 kHz with the Intan RHD2000 system (Intan Technologies), with a 50 Hz notch filter. All acquired data were then bandpass filtered between 20 and 500 Hz using custom MATLAB software before further analysis. In order to quantify the EUS EMG responses evoked by a single biphasic pulse of stimulation (30 repeated pulses at 1 Hz for each set of parameters), the peak-to-peak values of the evoked compound muscle action potential (CMAP) within 50 ms from the stimulation artifact was detected. The latency of the first evoked CMAP from the stimulation artifact was detected by thresholding above 3 standard deviations of the signal within the time window of 5–15 ms after the artifact. At longer phase widths or higher stimulation amplitudes, prolonged or multiple muscle potentials were also observed after the initial response. In order to quantify these prolonged responses, the EMG signals were rectified, and the area of the curve during a time window of 5–100 ms after stimulation onset was calculated. For quantification of EMG responses during the 10 Hz, 5-s-long stimulation, with or without high frequency blocking, the EMG signals were first processed for artifact removal (see Supplementary Figure 1).

### Statistical analysis

All statistical analyses were performed either using MATLAB or OriginPro (OriginLab, MA, USA). For the one-way repeated measures ANOVA test for multiple group comparisons, the *t*-test with Bonferroni correction was used as the *post-hoc* test. Alpha value used for all the statistical tests was 0.05. Data shown were mean ± standard error of mean (S.E.M.) unless indicated otherwise. The Kolmogorov–Smirnov test (OriginPro) was used to test for normality in the data distributions (with a criterion of *p* > 0.05) prior to using parametric statistical tests. Effect sizes for paired *t*-tests and one-way repeated measures ANOVA were calculated based on Hedges' *g* and η^2^ (eta squared), respectively (Lakens, 2013).

## Results

### Graded control of bladder contraction via low frequency pelvic nerve stimulation

**Figure 1:**
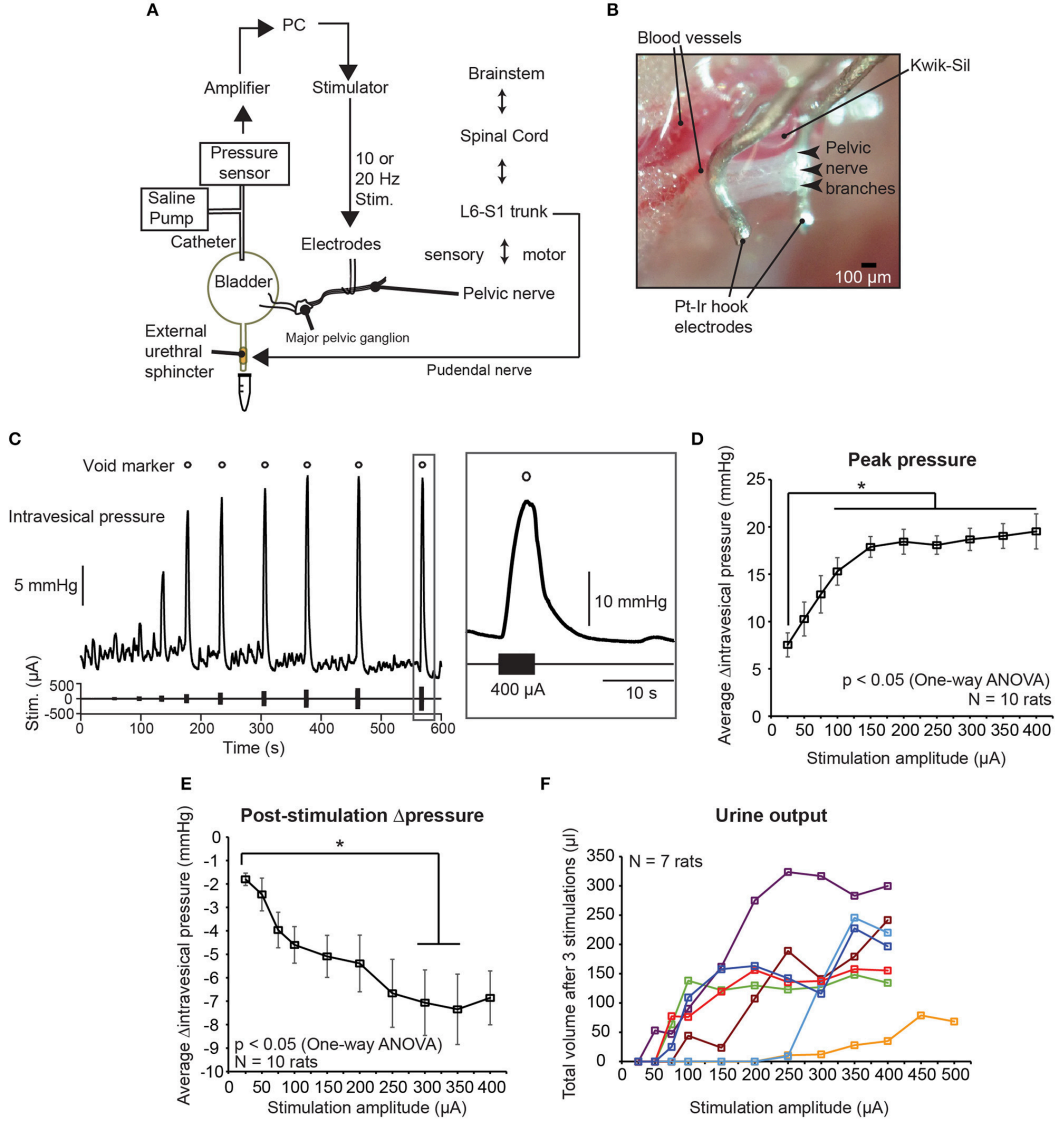
Graded control of bladder voiding by low frequency electrical stimulation of the pelvic nerve. **(A)** Experimental setup of unilateral stimulation of the pelvic nerve, and simultaneous intravesical pressure recording using anesthetized female rats. **(B)** Photomicrograph of platinum-iridium (Pt-Ir) electrodes interfacing with pelvic nerve branches and encapsulated with biological silicone elastomer (Kwik-Sil). **(C)** Example of graded intravesical pressure changing with increasing current amplitude used for stimulation. Each stimulation epoch was comprised of a 5-s-long burst of 10 Hz biphasic pulses. Open circles indicate stimulation epochs that resulted in bladder voiding. **(D)** Stimulation-evoked peak changes in intravesical pressure significantly increased with stimulation amplitude, and reached a plateau at higher amplitudes (one-way repeated measures ANOVA: *p* < 0.001, η^2^ = 0.451, *N* = 10 rats). The asterisk indicates that increases in intravesical pressure for stimulation amplitudes larger than 100 μA were significantly different from those obtained with 25 μA, *p* < 0.05 with the Bonferroni *post-hoc* test. Values are mean ± S.E.M. **(E)** Negative change in pressure after stimulation-evoked voiding became larger at higher stimulation amplitudes (one-way repeated measures ANOVA: *p* = 0.0019, η^2^ = 0.245, *N* = 10 rats). The asterisk indicates that decreases in intravesical pressure for stimulation amplitudes 300 and 350 μA were significantly different from those obtained with 25 μA, *p* <0.05 with the Bonferroni *post-hoc* test. Values are mean ± S.E.M. **(F)** Total volume of urine voided after three repeats of pelvic nerve stimulation at various current amplitudes (*N* = 7 rats). For 1 rat, stimulation current was increased up to 500 μA.

**Figure 2:**
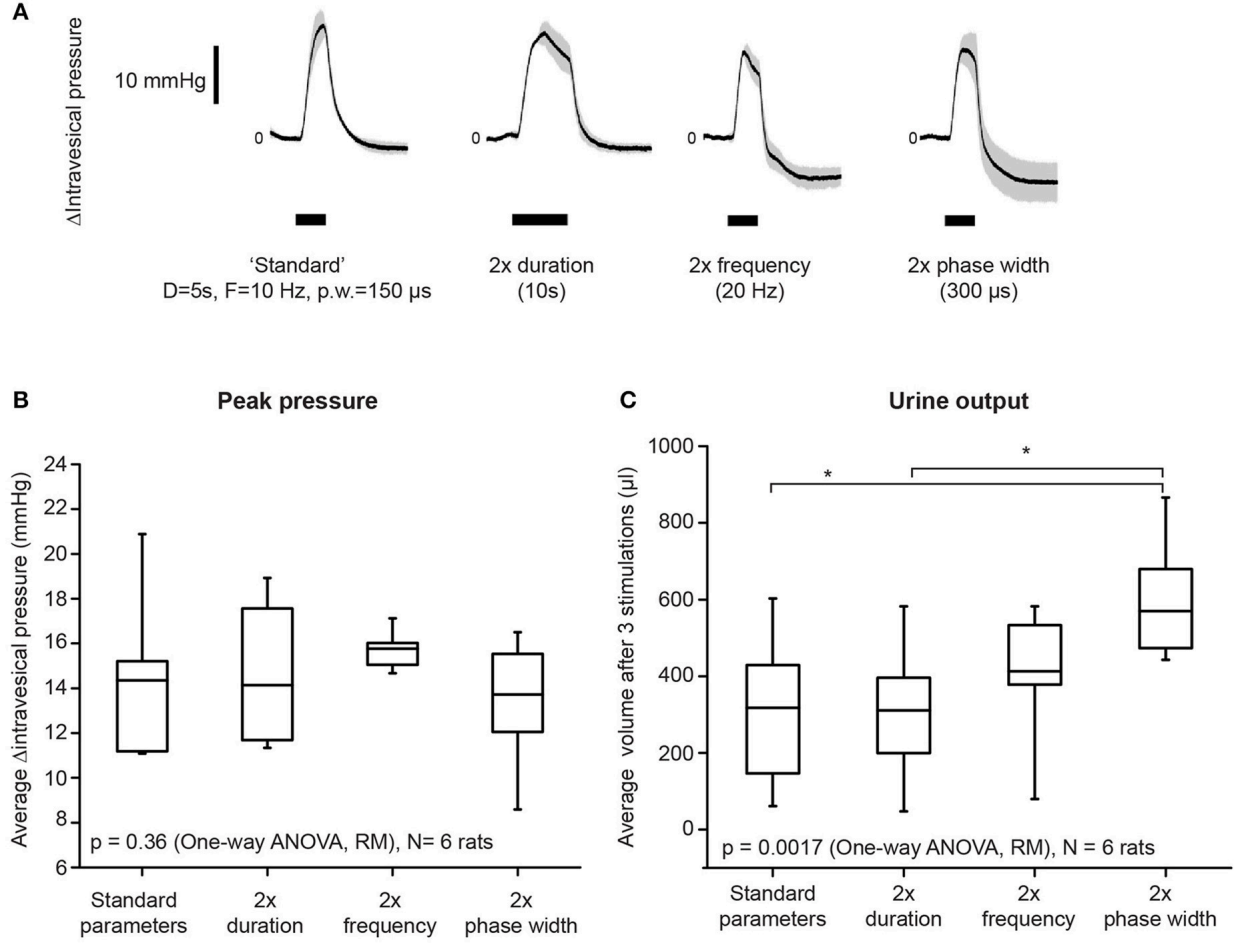
Increasing stimulation phase width had the strongest effect on improving bladder voiding**. (A)** Examples of average intravesical pressure changes in response to doubling either stimulation duration, frequency, or phase widths. The “standard” parameters used were: duration = 5 s, frequency = 10 Hz, and phase width = 150 μs. Current amplitudes used were suprathreshold (i.e., resulted in voiding), and were kept consistent within each rat. Shaded regions indicate standard deviations. **(B)** Average stimulation-evoked peak intravesical pressures were not significantly different when either duration, frequency, or phase widths were doubled (one-way repeated measures ANOVA: *p* = 0.36, *N* = 6 rats). The box indicates mean and 25th−75th percentile. **(C)** Doubling phase width resulted in significantly increased volume of urine voided, when compared to both standard parameters and duration = 10 s (one-way repeated measures ANOVA: *p* = 0.0017, η^2^ = 0.626, *N* = 6 rats). Asterisks indicate *p* < 0.05, with Bonferroni *post-hoc* test. Urine output was quantified as the total volume of urine voided after three repeated stimulation epochs for each condition. The box indicates mean and 25th−75th percentile.

In order to determine suitable parameters for the low frequency electrical stimulation of pelvic nerves to elicit voiding, we first stimulated pelvic nerve branches, central to the MPG, unilaterally in anesthetized female rats with different stimulation parameters, such as current amplitude, frequency, duration, and phase widths, and examined intravesical pressure changes and voiding outcomes (Figures 1A,B, 2A). The current amplitudes used in our experiments fell within normal ranges of nerve stimulation associated with lower urinary tracts (e.g., pudendal nerve; Boggs et al., 2005, 2006), and were able to elicit graded bladder contractions, as well as voiding at higher, suprathreshold amplitudes (Figure 1C). Intravesical pressure increased with stimulation currents at lower values, but saturated at larger current amplitudes (Figure 1D, one-way repeated measures ANOVA: *p* < 0.001, η^2^ = 0.451, followed by Bonferroni *post-hoc* test: *p* < 0.05, *N* = 10 rats). Furthermore, post-stimulation decreases in intravesical pressure, which typically occurred due to voiding, showed significantly larger pressure drops at higher stimulation current amplitudes (Figure 1E, one-way repeated measures ANOVA: *p* = 0.0019, η^2^ = 0.245, followed by Bonferroni *post-hoc* test: *p* < 0.05, *N* = 10 rats). However, urine output was highly variable across rats, with currents required for suprathreshold voiding responses ranging from 50 to 250 μA (Figure 1F, *N* = 7 rats, voiding outcomes were not measured for the other 3 rats).

Doubling either the stimulation frequency (from 10 to 20 Hz), duration (from 5 to 10 s), or phase width (from 150 to 300 μs) did not significantly increase intravesical pressure (Figure 2B, *p* = 0.36, one-way repeated measures ANOVA, *N* = 6 rats). However, doubling the phase width, but not duration and frequency, significantly increased urine output (Figure 2C, one-way repeated measures ANOVA: *p* = 0.0017, η^2^ = 0.626, followed by Bonferroni *post-hoc* test: *p* < 0.05, *N* = 6 rats). These results established the appropriate range of stimulation parameters that successfully evoked voiding responses through unilateral pelvic nerve stimulation.

### Stimulation of distally transected pelvic nerve removes bladder-sphincter dyssynergia

**Figure 3:**
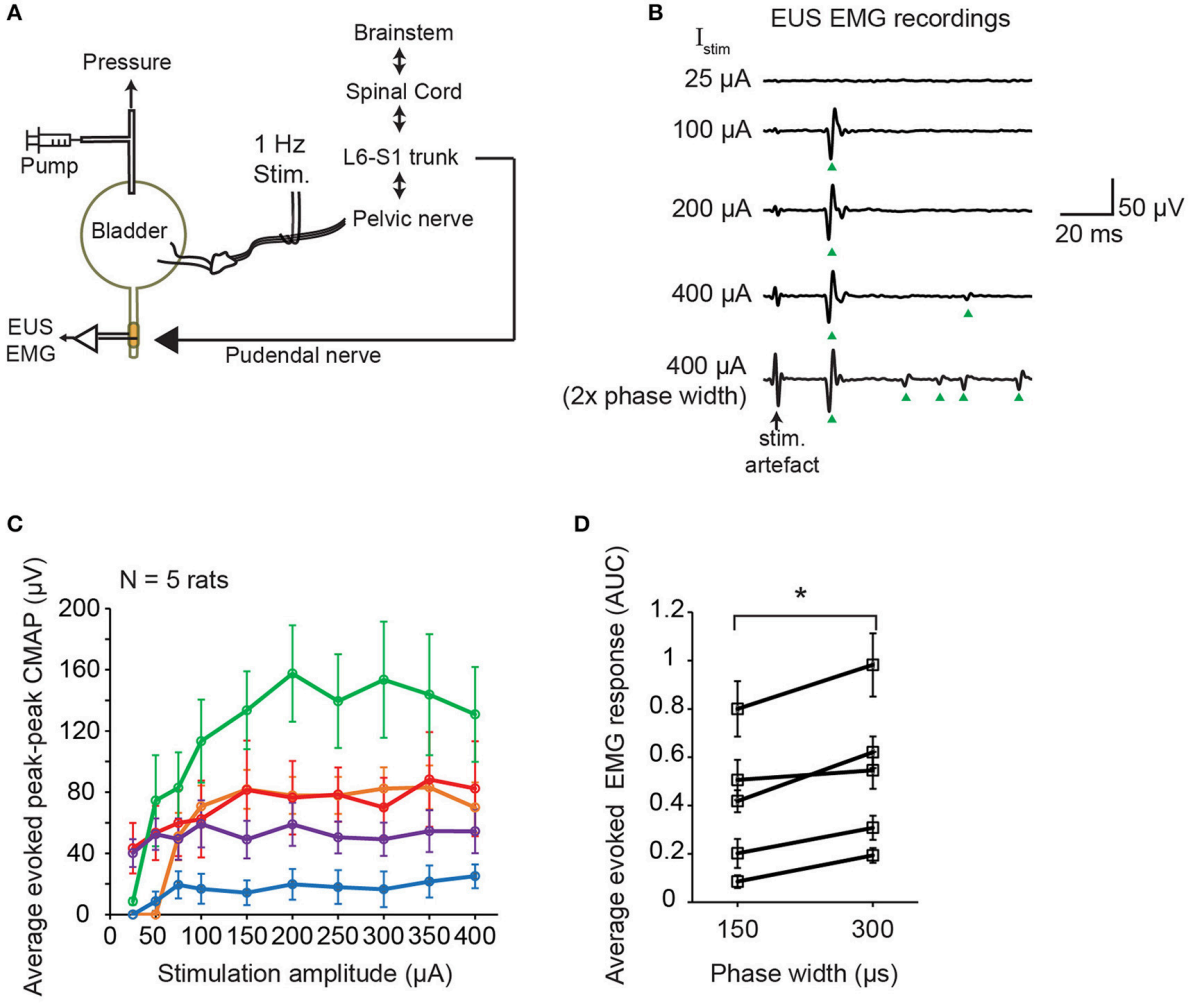
Single pulse stimulation of pelvic nerve evoked EUS muscle contractions**. (A)** Schematic diagram of simultaneous electrical stimulation of the pelvic nerve and electromyography (EMG) of the external urethral sphincter (EUS) in anesthetized female rats. **(B)** Examples of evoked EUS muscle activity in response to increasing current amplitudes and phase widths of single biphasic pulse stimulation of the pelvic nerve (delivered at 1 Hz). Green arrowheads indicate visible compound muscle action potentials (CMAP). **(C)** Average evoked peak-to-peak CMAP responses to increasing stimulation current amplitudes across 5 rats (*n* = 30 stimulation trials for each condition). **(D)** Average evoked EMG response, quantified as the area under the curve (AUC) of the rectified EMG response (time window: 5–100 ms), significantly increased when stimulation pulse width increased from 150 to 300 μs, with current amplitude fixed at 400 μA (paired *t*-test: *p* = 0.012, Hedges' *g*: 1.76, *N* = 5 rats). The asterisk indicates *p* < 0.05 (paired *t*-test).

Timely relaxation of the EUS muscles during contraction of the bladder detrusor muscle is required for successful voiding. In order to elucidate the immediate effect of pelvic nerve electrical stimulation on the EUS muscle, we performed electromyography (EMG) of the EUS muscles while stimulating the pelvic nerve using single biphasic pulses delivered at 1 Hz (Figure 3A). Distinct compound muscle action potentials (CMAP) were detected following the stimulation artifacts of the single biphasic pulses (Figure 3B). In some cases, at higher stimulation current amplitudes, muscle potentials were also observed at later latencies after the initial CMAP response (Figure 3B). Across animals, the magnitudes of the peak-to-peak CMAP evoked by the same stimulation currents were highly variable (Figure 3C). The initial CMAP responses showed qualitative increases to small rises in stimulation amplitudes, but saturated at higher amplitudes (Figure 3C, *N* = 5 rats). The increases in stimulation phase width which was shown to increase urine output (Figure 2C), also led to stronger evoked EMG responses (Figure 3D, paired *t*-test: *p* = 0.012, Hedges' g: 1.76, *N* = 5 rats). Despite variability in evoked CMAP magnitude, the latency of the initial CMAP did not significantly vary, across animals, between smaller and larger stimulation amplitudes: for 75 μA, the latency was 19.9 ± 1.3 ms (mean ± S.E.M.), while at 400 μA, the latency was 20.5 ± 1.5 ms (mean ± S.E.M.), respectively (*p* = 0.78, paired *t*-test).

**Figure 4:**
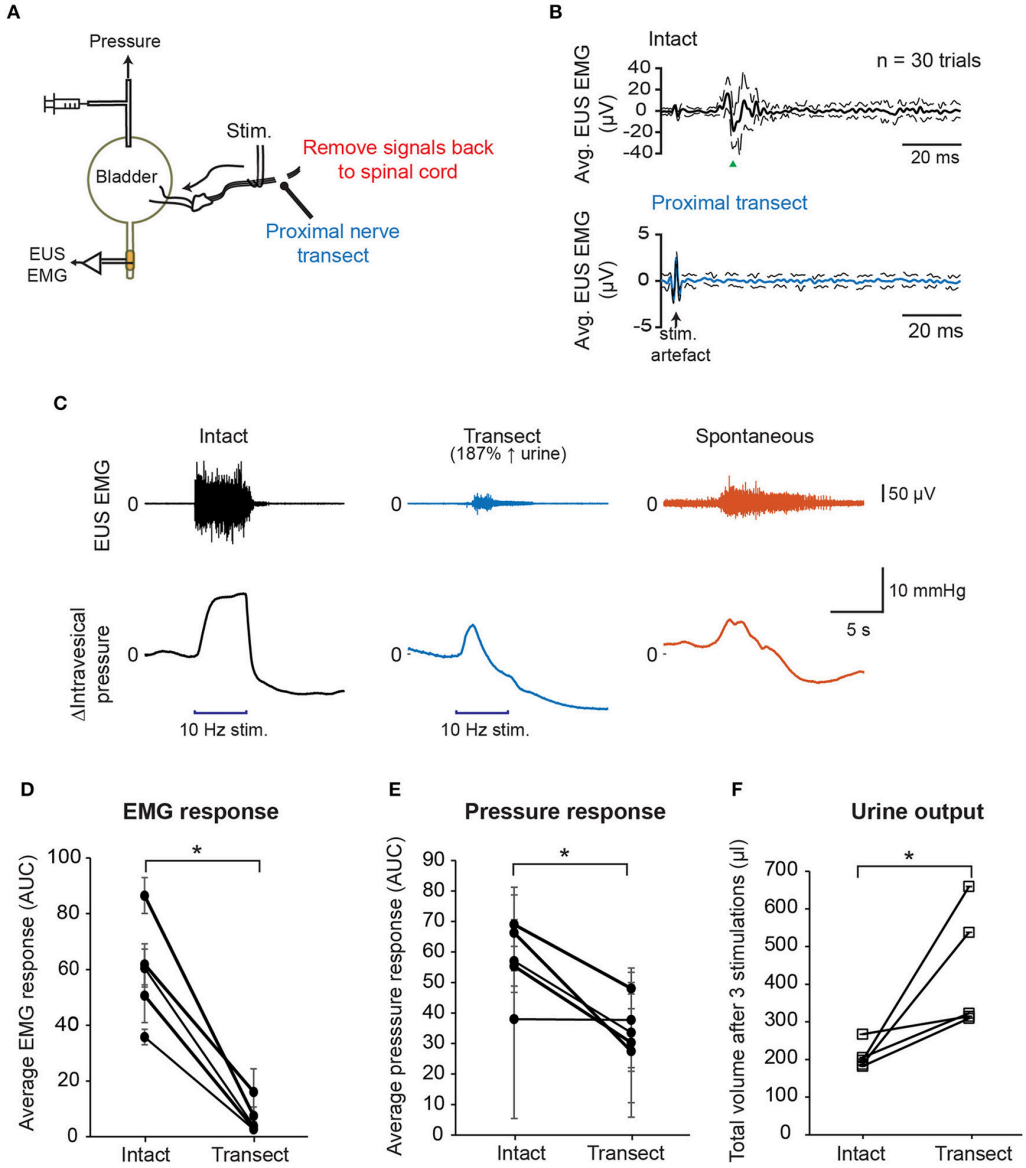
Disruption of the afferent nerve signals sent to the spinal cord by nerve transection reduced EUS muscle activity and improved voiding outcomes. **(A)** Schematic diagram showing transection of the pelvic nerve proximal to the site of stimulation. **(B)** Proximal nerve transection removed short latency stimulation-evoked EUS muscle activity. Example of averaged EMG recordings when the nerve was intact (black solid line, top panel) and transected (blue solid line, bottom panel), respectively, using the same stimulation parameters: current = 400 μA, phase width = 300 μs, *n* = 30 trials. Dashed lines indicate the standard deviation. **(C)** Examples of EUS EMG recordings and intravesical pressure responses to either 5 s-long stimulation of the intact pelvic nerve (black traces), stimulation of the proximally transected pelvic nerve (blue traces), or spontaneous bladder-filling related voiding (orange traces). **(D)** Average EMG response evoked by 10 Hz, 5 s stimulation, quantified as area under the curve, or AUC, of the rectified, low-pass-filtered EMG, significantly decreased after nerve transection (paired *t*-test: *p* = 0.002, Hedges' *g*: 2.77, *N* = 5 rats). **(E)** Positive changes in intravesical pressure, quantified as AUC of the intravesical pressure curve during nerve stimulation, significantly decreased after nerve transection (paired *t*-test: *p* = 0.025, Hedges' *g*: 1.42, *N* = 5 rats). **(F)** Stimulation-driven urine output increased significantly after proximal nerve cut (paired *t*-test: *p* = 0.049, Hedges' *g*: 1.13, *N* = 5 rats). Total urine volume was measured after three repeated nerve stimulations for each nerve condition. The asterisk indicates *p* < 0.05 (paired *t*-test).

Next, we tested whether propagation of the action potentials of the stimulated pelvic nerve fibers back to spinal cord is indeed the main route for evoking the observed CMAP of the EUS. We performed nerve stimulation with a proximal nerve transection to remove any direct nerve inputs back to the spinal cord and measured intravesical pressure, EUS EMG, and urine output (Figure 4A). With the proximal nerve cut, single biphasic pulse stimulation no longer evoked the CMAP typically observed with stimulation of the intact pelvic nerve (Figure 4B). This result indicated that intact nerve conduction to the spinal cord was required for pelvic nerve stimulation to recruit neural pathways that subsequently lead to pudendal nerve activation, and undesirable stimulation-evoked bladder-sphincter dyssynergia.

In order to examine how proximal nerve transection impacted stimulation-evoked bladder contractions and voiding outcomes, we stimulated both intact and transected nerves distally at 10 Hz for 5 s at 400 μA (Figure 4C). EUS EMG signals were first processed to remove stimulation artifacts (see Supplementary Figure 1). Overall, the average evoked EUS EMG responses decreased significantly when the pelvic nerve was transected (Figure 4D, paired *t*-test: *p* = 0.002, Hedges' g: 2.77, *N* = 5 rats, 3 trials each). Remnant EUS muscle activity was still observed in some stimulation trials after unilateral proximal nerve cut (Figure 4C and see Supplementary Figure 2), and this was likely due to the presence of intact sensory reflex pathways in the other pelvic nerve, or through urethral sensory fibers. Peak bladder pressures were also less sustained in the transected nerve condition compared to the condition with intact nerves, and more similar to intravesical pressure changes during spontaneous (non-stimulated) voiding (Figure 4C). The rise in intravesical pressure decreased significantly in the transected condition compared to the intact nerve condition (Figure 4E, paired *t*-test: *p* = 0.025, Hedges' g: 1.42, *N* = 5 rats, 3 trials each). The baseline pressure signals were not significantly different between trials with intact and transected nerves (see Supplementary Figure 2, *p* = 0.393, paired *t*-test, *N* = 5). Urine output during stimulation also increased significantly after the proximal nerve cut compared to when the nerve was intact (Figure 4F, paired *t*-test: *p* = 0.049, Hedges' g: 1.13, *N* = 5 rats). These results indicated that pelvic nerve stimulation-evoked voiding did not depend on the excitation of the sensory reflex pathway, but instead recruited the parasympathetic efferent pathway directly. In addition, complete blocking of the sensory signals leading back to the spinal cord evoked by the pelvic nerve stimulation reduced significantly EUS activity, and increased voiding output.

### High frequency stimulation of pelvic nerves generated brief onset effects at lower current amplitudes

**Figure 5:**
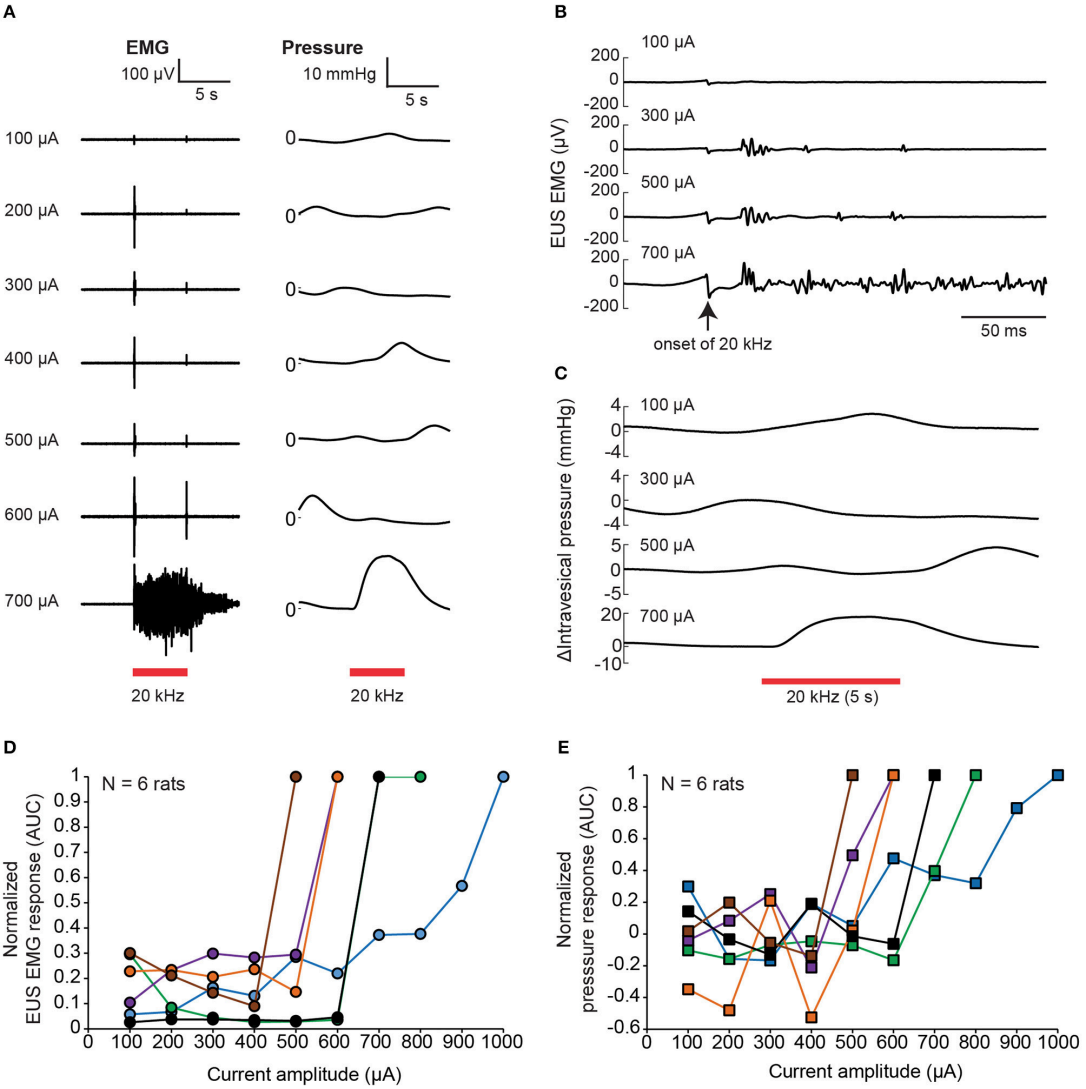
Brief EUS EMG onset effects associated with kHz frequency electrical stimulation (KHFS) of the pelvic nerve. **(A)** EUS EMG recordings and intravesical pressure changes during application of increasing (100 μA steps) current amplitudes of 20 kHz electrical stimulation to the pelvic nerve in a single rat. **(B)** Evoked EUS EMG effects were brief at lower current amplitudes, and sustained at high current amplitudes of the 20 kHz electrical stimulation to the pelvic nerve (zoomed-in view of data as shown in **A**). **(C)** Intravesical pressure responses during the 20 kHz stimulation were slower and harder to distinguish from spontaneous fluctuations at lower current amplitudes. **(D)** Summary of normalized EMG responses evoked during KHFS against current amplitude used (*N* = 6 rats, each signal was normalized to the corresponding maximum value obtained for each rat). **(E)** Summary of normalized pressure changes evoked during KHFS against current amplitude used (*N* = 6 rats, each signal was normalized to the corresponding maximum value obtained for each rat).

The nerve transection experiment demonstrated that voiding output can be increased by preventing inadvertent afferent sensory recruitment from evoking EUS contractions. This could also be done using nerve-blocking agents such as lidocaine. On the other hand, the alternative technique of KHFS to prevent dyssynergia had not been attempted for these thin pelvic nerve branches. Thus, we first examined the effect of 20 kHz electrical stimulation of pelvic nerve alone on EUS EMG and intravesical pressure with rectangular, biphasic pulses with 100 μA incremental steps (Figure 5A). Brief EUS EMG responses were typically detected after the onset of the 20 kHz stimulation at lower current amplitudes, with the responses adapting or disappearing before the end of the 5-s stimulation period (Figure 5B). The initial evoked EUS EMG responses were likely due to brief nerve activation preceding the block of nerve conduction, and those responses adapted or disappeared as the effects of nerve conduction block set in (Kilgore and Bhadra, 2014). However, beyond a certain high current “excitatory threshold,” sustained and tonic EMG activity that lasted throughout the stimulation period was observed (see 700 μA; Figure 5A). The sustained EMG activity was also accompanied with noticeable increases in intravesical pressure (Figures 5A,C), which could be caused by direct excitation of the efferent pathway to the bladder, or indirectly by prolonged closure of the EUS muscle, or a combination of both possibilities. The value of the high current “excitatory threshold” varied between experiments, and across rats (Figures 5D,E, *N* = 6 rats). Hence, the threshold was first established for each rat before subsequent testing. Further nerve conduction block experiments were limited to current amplitudes below this threshold.

### High frequency conduction block of pelvic nerves depends on the ratio of blocking to stimulation current amplitude

**Figure 6:**
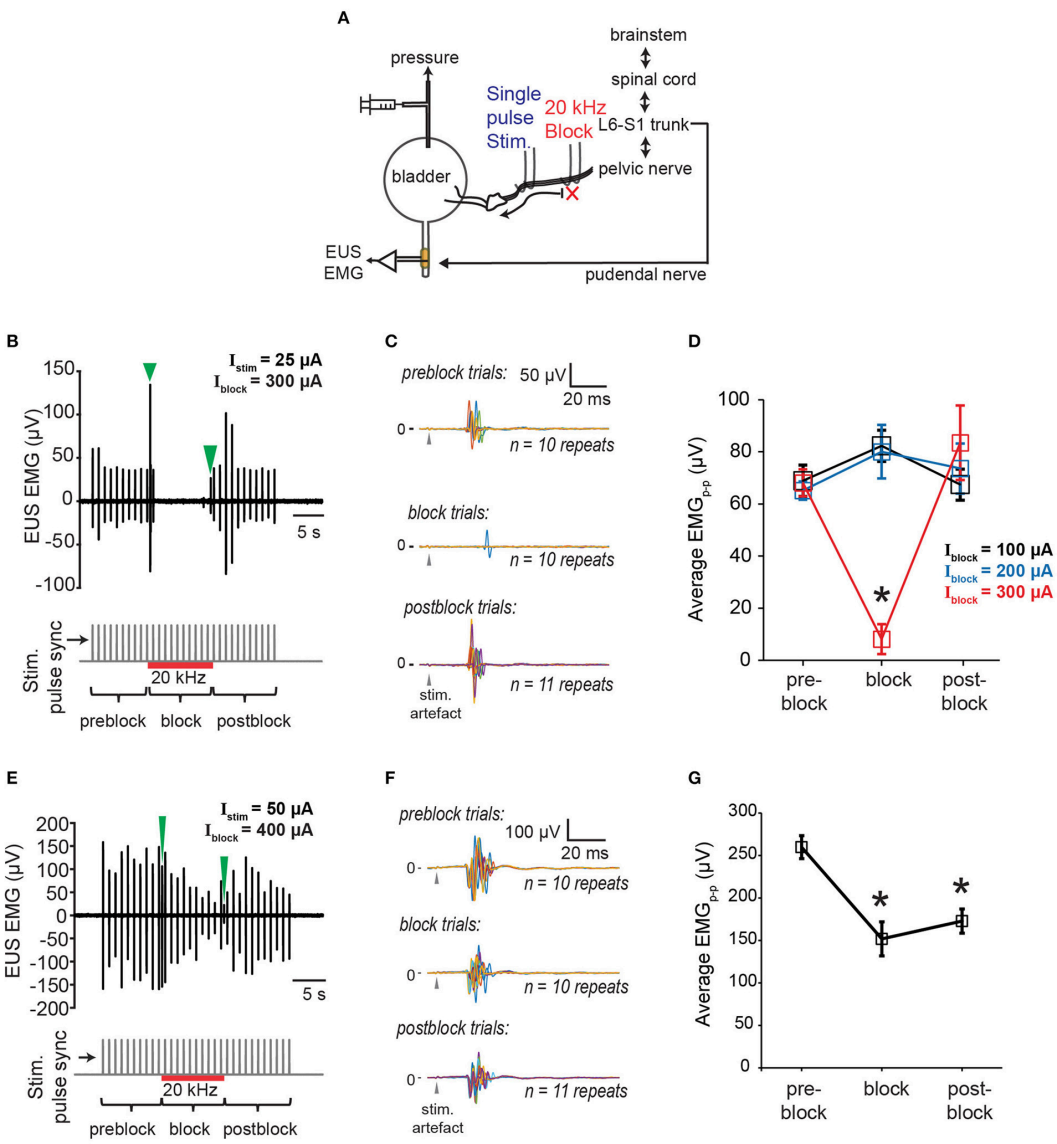
Blocking of EUS contractions evoked by single pulse pelvic nerve stimulation**. (A)** Schematic of experimental setup for testing efficacy of proximal KHFS to prevent activation of EUS during single pulse stimulation of the pelvic nerve. **(B)** Example of immediate and reversible block of EUS contractions evoked by single pulse stimulation (stimulation current amplitude, I_stim_ = 25 μA) of the pelvic nerve during application of 20 kHz electrical block (block current amplitude, I_block_ = 300 μA). Green arrowheads indicate block related onset and offset signals in the top plot. Stimulation onset, and the period of the high frequency block are indicated by gray vertical lines and the red horizontal line, respectively, in the bottom plot. **(C)** The data in **(B)** are shown as superimposed EUS EMG traces within a 100 ms window from the stimulation onset (gray arrowheads), obtained during the pre-block, block, and post-block periods, respectively (*n* = 10–11 repeats for each period). **(D)** Average peak-to-peak evoked EUS EMG (EMGp-p) during pre-block, block, and post-block periods for I_block_ of 100, 200, and 300 μA. The asterisk indicates *p* < 0.05 (one-way repeated measures ANOVA: *p* < 0.0001, η^2^ = 0.544, followed by Bonferroni *post-hoc* test compared to the pre-block period). **(E)** Example of partial block and partial recovery of EUS contractions (I_stim_ = 50 μA; I_block_ = 400 μA). Green arrowheads indicate block related onset and offset signals in the top plot. Stimulation onset, and the period of the high frequency block are indicated by gray vertical lines and the red horizontal line, respectively, in the bottom plot. **(F)** The data in **(E)** are shown as superimposed EUS EMG traces within a 100 ms window from the stimulation onset (gray arrowheads), obtained during the pre-block, block, and post-block periods, respectively (*n* = 10–11 repeats for each period). **(G)** Average peak-to-peak evoked EUS EMG (EMGp-p) during pre-block, block, and post-block periods for I_stim_ of 50 μA and I_block_ of 400 μA. Asterisks indicate *p* < 0.05 (one-way repeated measures ANOVA: *p* = 0.0001, η^2^ = 0.479, followed by Bonferroni *post-hoc* test compared to the pre-block period).

**Figure 7:**
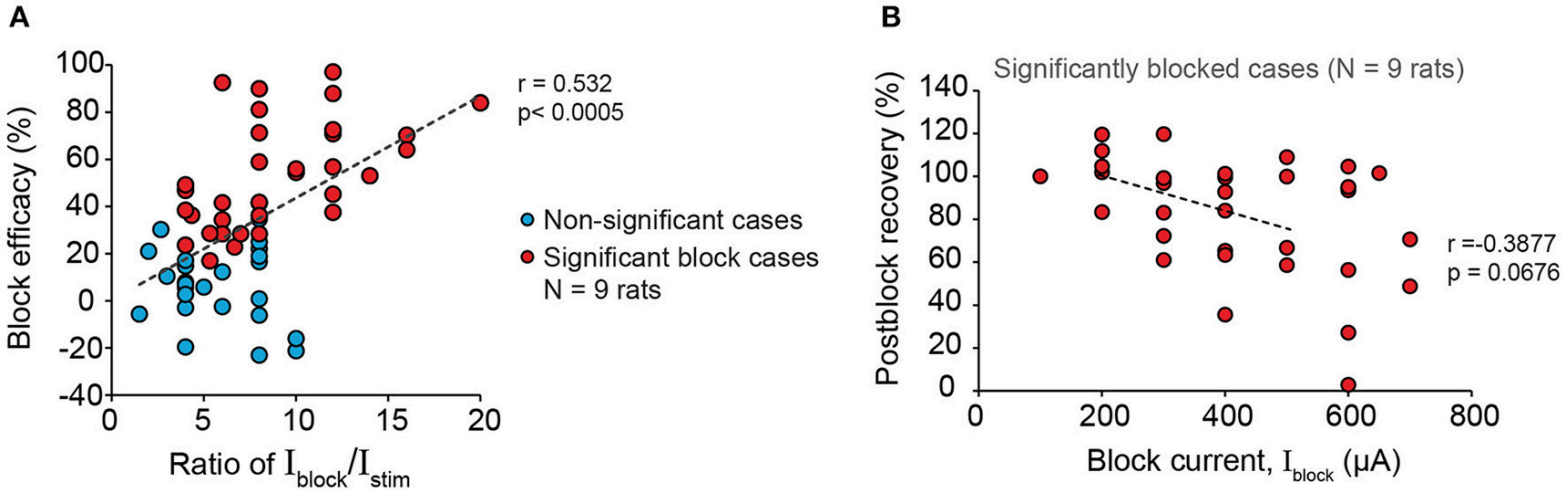
Larger blocking to stimulation current amplitude ratio increases efficacy of blocking EUS contractions during pelvic nerve stimulation. **(A)** Block efficacy correlated positively with the I_block_/I_stim_ ratio (Pearson's linear correlation coefficient, *r* = 0.532, *p* < 0.0005, *N* = 9 rats). Block efficacy was measured as the percentage reduction of evoked EMGp-p during block compared to the pre-block period. **(B)** Post-block recovery was not significantly correlated to the amplitude of the blocking current within the range of 200–500 μA (Pearson's linear regression, *r* = −0.3877, *p* = 0.0676, dashed line, 100, 600, and 700 μA were excluded due to the limited number of data points and the presence of heteroscedasticity). Post-block recovery was measured by the percentage of evoked EMGp-p during the post-block period compared to that during the pre-block period.

Next, we examined the effectiveness of proximal 20 kHz stimulation to block evoked EUS CMAP during a single biphasic pulse (phase width = 150 μs) stimulation, delivered at 1 Hz to the pelvic nerve (Figure 6A). During the 30 s of 1 Hz stimulation, EUS EMG was recorded continuously before, during, and after a 10-s period of a 20 kHz block (Figure 6B). When the stimulation current (I_stim_) was much lower than the current used for the 20 kHz block (I_block_), an almost immediate and reversible block of the evoked EUS activity could be observed (Figures 6B,C), demonstrating that sensory action potentials on their way to the spinal cord were being effectively blocked by the proximal KHFS application. In order to gain a better understanding of the relationship between I_stim_ and I_block_, we altered the two currents systematically, increasing I_stim_ by 25–50 μA steps. I_block_ was incrementally increased by 100 μA until a significant decrease in the evoked CMAP was observed (Figure 6D, one-way repeated measures ANOVA: *p* < 0.0001, η^2^ = 0.544, followed by Bonferroni *post-hoc* test compared to the pre-block period: *p* < 0.05). However, in many cases, partial block and partial post-block recovery of EUS were also observed (Figures 6E–G), in addition to the occasional complete failure to block. Overall, we found that the blocking efficacy correlated positively with the ratio of I_block_/I_stim_ (Figure 7A, linear regression, *r* = 0.532, *p* < 0.005, dashed line, *N* = 9 rats). Although qualitatively there was a decreasing trend in the post-block recovery with increasing blocking current, the post-block recovery was not significantly correlated with the amplitude of the blocking current, I_block_, from 200 to 500 μA range (Figure 7B, Pearson's linear regression, *r* = −0.3877, *p* = 0.0676, dashed line, 100, 600, and 700 μA were excluded due to a small number of data points, and the existence of heteroscedasticity). These results suggest that immediate and reversible blockage of evoked EUS CMAP could be achieved more effectively by using the lowest current amplitude necessary for the low frequency stimulation to evoke bladder voiding, which would allow lower current amplitudes for KHFS to be used.

### Combining distal low frequency stimulation and proximal KHFS block of the pelvic nerve reduced bladder-sphincter dyssynergia

**Figure 8:**
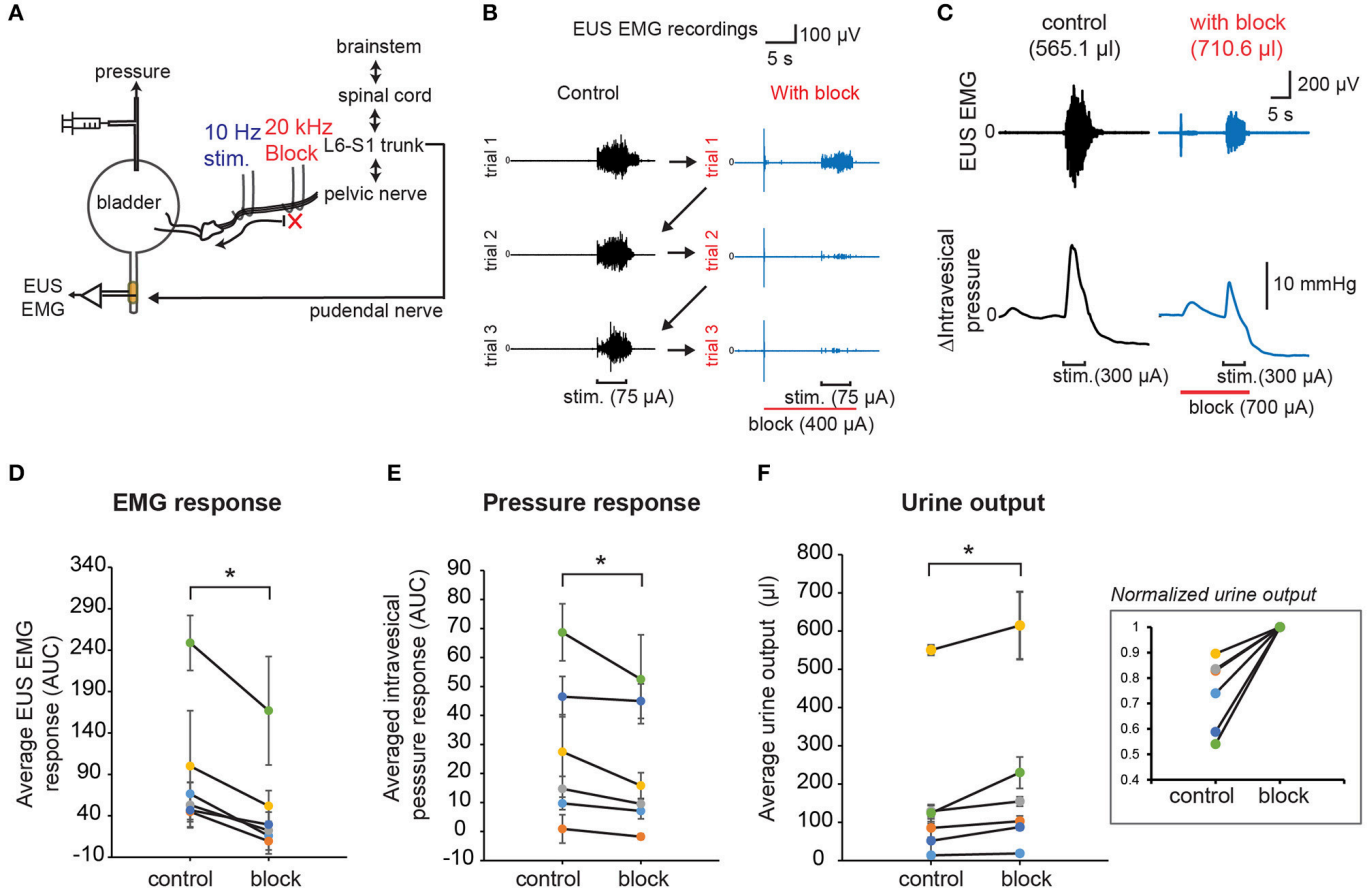
KHFS block combined with low frequency stimulation of pelvic nerves improved voiding outcomes and reduced bladder-sphincter dyssynergia. **(A)** Schematic diagram of combining proximal KHFS (20 KHz) with distal low frequency (10 Hz) stimulation of pelvic nerves to activate specifically efferent pathway to the bladder. **(B)** EUS EMG recordings obtained during control trials (10 Hz stimulation only; black traces) interleaved with blocking trials (10 Hz stimulation with 20 kHz block; blue traces) in a rat. Stimulation and block periods, together with current amplitudes used, are indicated below the EMG traces (stimulation period = 5 s and block period = 16 s). Arrows indicate the sequence of interleaved repeated control and block trials. **(C)** Example of simultaneously recorded EUS EMG and intravesical pressure changes during control (black traces) or with 20 kHz block (blue traces) trials. Stimulation and block periods and amplitudes are indicated below the pressure graphs. Corresponding urine output were indicated in parentheses above the EMG traces. **(D)** Average stimulation-evoked EUS EMG responses (measured as area under curve of rectified, integral EMG during the stimulation period) significantly decreased in presence of proximal high frequency block (paired *t*-test: *p* < 0.0005, Hedges' *g*: 1.15, *n* = 6 cases with 3 repeats each, *N* = 4 rats). **(E)** Average stimulation-evoked intravesical pressure changes (measured as area under curve during the stimulation period, AUC) significantly decreased in presence of proximal high frequency block (paired *t*-test: *p* = 0.0139, Hedges' *g*: 0.63 *n* = 6 cases with 3 repeats each, N = 4 rats). **(F)** Average stimulation-evoked urine output significantly increased in presence of proximal high frequency block (paired *t*-test: *p* = 0.00189, Hedges' *g*: 0.84, *n* = 6 cases with 3 repeats each, N = 4 rats). The inset shows normalized values to the maximum mean urine output obtained for each case. The asterisk indicates *p* < 0.05 (paired *t*-test).

In order to demonstrate the effect of combining low frequency stimulation with high frequency block on stimulation-evoked bladder-sphincter dyssynergia and voiding outcomes, we performed unilateral 10 Hz stimulation with proximal 20 kHz block on the pelvic nerve branches while measuring EUS EMG, intravesical pressure, and urine output (Figure 8A). As opposed to the permanent block imposed by the proximal nerve transection experiments shown in Figure 4, the reversibility of the 20 kHz block allowed us to perform interleaved trials between control experiments (low frequency stimulation only) and block experiments (low combined with kilohertz frequency stimulation) for 3 repeats each within a subject (Figure 8B). Suprathreshold current amplitudes that evoked voiding responses were used for the 10 Hz stimulation, while blocking amplitudes below the “excitatory threshold” that still exhibited significant blocking of EUS CMAP evoked by the single pulse stimulation were used for the 20 kHz block (parameters used for the different rats are shown in Supplementary Table 1). In order to isolate the onset effects of 20 kHz block on EUS EMG from that evoked by 10 Hz stimulation, and to allow the effect of nerve conduction block to set in, the block was started 10 s prior to the start of the 10 Hz stimulation, and lasted 1 s past the end of the low frequency stimulation (Figure 8C).

Overall, the average evoked EUS EMG response (Figure 8D, paired *t*-test: *p* < 0.0005, Hedges' *g*: 1.15, *n* = 6 cases with 3 repeats each, *N* = 4 rats) and intravesical pressure response (Figure 8E, paired *t*-test: *p* = 0.0139, Hedges' *g*: 0.63 *n* = 6 cases with 3 repeats each, *N* = 4 rats) significantly decreased in block trials compared to control trials, indicative of reduced bladder-sphincter dyssynergia. Importantly, the average urine output also increased significantly in block trials (Figure 8F, paired *t*-test: *p* = 0.00189, Hedges' *g*: 0.84, *n* = 6 cases with 3 repeats each, *N* = 4 rats). The average increase in urine output in block trials compared to control trials was 40.5 ± 12.3% (*n* = 6 cases in 4 rats). The baseline pressure signals were not significantly different between the interleaved control and block trials (see Supplementary Figure 3A, *p* = 0.257, paired *t*-tests, *n* = 6 cases in 4 rats). A closer look at the EUS EMG response in consecutive trials (see Supplementary Figure 3B) suggested that there were “carry-over” effects that reduced the EUS EMG response in the second and third control trials due to partial reversibility of the 20 kHz block (see Figures 6E, 7B). Despite the carry-over effects, pressure responses were decreased and urine output were increased in block trials compared to control trials (Figures 8E,F and Supplementary Figures 3C,D). Without these “carry-over” effects, the reduction in EUS EMG response, pressure response, and increase in urine output when comparing the control to the block trials may have been even larger. These results demonstrate that combining low frequency stimulation and KHFS block of pelvic nerves can be successfully applied to reduce bladder-sphincter dyssynergia for improved bladder voiding outcomes.

## Discussion

This study is the first to perform combined low frequency stimulation and kilohertz frequency stimulation (KHFS) of the pelvic nerve to evoke efficient micturition by overcoming bladder-sphincter dyssynergia. This novel stimulation paradigm activated the parasympathetic efferent pathway to achieve graded control of the bladder, while reversibly blocking unwanted afferent activation, thus resulted in improved voiding with reduced sphincter activity. Our results also demonstrate the technical feasibility of achieving such a stimulation paradigm in fine autonomic mixed nerves while limiting the length (a few mm) of the exposed nerve. Importantly, our findings could be useful for advancing pelvic nerves as a more suitable neuromodulation target for restoring voluntary control of bladder voiding in the future.

The stimulation parameters used in our study produced graded recruitment of the parasympathetic efferent population present in the pelvic nerve branches (see Figure 1). As our stimulation electrodes were placed on the pelvic nerve lateral and central to the MPG, we are likely to be stimulating parasympathetic pre-ganglionic fibers originating from the spinal intermediolateral nucleus (L6-S1 level in rats), which then synapse onto the pelvic post-ganglionic neurons in the MPG that excite the bladder. As increasing phase width had the strongest effect in improving voiding (Figure 2), this suggests that more effective activation of parasympathetic fibers was achieved with longer phase widths. Currently, the two known excitatory mechanisms of bladder contraction involve either acetylcholine release, or ATP release from the post-ganglionic axon terminals, which bind to M_3_ muscarinic receptors or P2X purinergic receptors present on detrusor muscles, respectively (Fowler et al., 2008). Future work could delineate if both neurotransmitter mechanisms are involved in producing the graded bladder contraction driven by our electrical stimulation.

However, other than the parasympathetic fibers, stimulation of the mixed pelvic nerve also activated the guarding reflex, and evoked bladder-sphincter dyssynergia. The sensory afferents of the pelvic nerve include both myelinated Aδ mechanoreceptive fibers and unmyelinated nociceptive C fibers. Normally, the sensory Aδ fibers are activated during bladder filling to generate the pelvic-to-pudendal reflex to preserve continence until a void threshold is reached (Fowler et al., 2008). The approximately 19–20 ms latency of the first evoked EUS CMAP, and the presence of longer latency CMAPs observed at higher stimulation amplitudes (see Figure 3), are similar to values reported in a previous study performed on rats under urethane anesthesia. It has been postulated that both the short- and long-latency EUS activity arose from activation of Aδ fibers, and not C fibers (Chang, 2006). Despite the presence of bladder-sphincter dyssynergia, our finding that voiding was still observed with low frequency stimulation alone is due to sustained detrusor contractions and bladder pressures high enough, albeit undesirable, to overcome the urethral resistance.

Consistent with reduced bladder-sphincter dyssynergia, our results showed that combining low frequency stimulation and KHFS evoked less EUS muscle activity, decreased bladder pressure during voiding, and improved urine output (see Figure 8). This is similar to the improved voiding achieved by the non-reversible method of proximal nerve transection (see Figure 4). In contrast to previous studies that had reported lower voiding efficiency after synergistic EUS EMG bursting activity was disrupted by pudendal nerve transection (Peng et al., 2006; Cruz et al., 2014), our study indicates that the ability to reduce dyssynergic EUS contractions induced by pelvic nerve stimulation improves voiding as long as the detrusor muscles in the bladder are able to generate enough contractile forces to cause voiding.

While we cannot definitively conclude from our experiments that the 20 kHz stimulation applied proximally to the stimulation was sufficient to block unmyelinated C fibers or antidromic stimulation of parasympathetic efferents, the fact that short latency evoked EUS EMG responses were blocked by 20 kHz stimulation (Figure 6) suggests that it is likely that the nerve conduction block was at least induced in the Aδ fibers. As C fibers and parasympathetic fibers are typically smaller than Aδ fibers (Hulsebosch and Coggeshall, 1982; Schalow, 2009; Birder et al., 2010), a higher threshold current is likely to be required for both stimulation and nerve conduction block (Blair and Erlanger, 1933; Tai et al., 2005; Zhang et al., 2006b). Future work would involve spinalized rats with hyperreflexive bladder (Shaker et al., 2003) to elucidate if 20 kHz stimulation also blocks C fibers or parasympathetic fibers. Experiments in a spinalized model may also advance the usefulness of this technique for reducing neurogenic detrusor activity or hyperreflexia present in chronic spinal cord injured patients (which has been linked with neural plasticity revealing C fiber related reflexes, de Groat and Yoshimura, 2006, 2012). Being able to tackle different clinical models of bladder dysfunction by targeting the same nerve structure could make the approach appealing. While the work by Crook and Lovick (2017) showed that 1–3 kHz stimulation can prevent voiding in healthy rodents, their results indicate that the blocking mechanism works centrally. Thus, it remains unclear if their technique will also prevent unwanted contractions in subjects with spinal cord injury above the lumbosacral level.

Our stimulation approach and findings are distinct from that of SARS. In SARS, the sacral anterior root is stimulated with multiple intermittent pauses to allow the striated EUS muscles to relax, while the slower smooth detrusor muscles continue to contract. However, this stimulation paradigm can still lead to relatively high and sustained bladder pressures (Kirkham et al., 2002). Effectiveness in decreasing the bladder pressure during voiding is important to prevent damage to the upper urinary tract and vesicoureteral reflux (Dorsher and McIntosh, 2012). Importantly, improved urination will also reduce the post-void residual volume of the bladder, and thus prevent urinary tract infections. Thus, kilohertz frequency block of pelvic nerves is a more reversible method compared to dorsal rhizotomy (Van Kerrebroeck et al., 1996; Popovic, 2002) or sacral denervation (Hohenfellner et al., 2001b). Dorsal rhizotomy and sacral denervation, which have been shown to reduce autonomic dysreflexia, can lead to other irreversible side effects, such as reduced sexual function or bowel control.

Our approach of combining 20 kHz stimulation with low frequency stimulation is also different compared to the technique of anodal block that has been used to achieve selective activation of smaller parasympathetic fibers over larger somatic fibers in sacral nerves (Brindley and Craggs, 1980; Rijkhoff et al., 1994; Vučković and Rijikhoff, 2004). Anodal block has been used to reduce detrusor-sphincter dyssynergia during intra-operative testing in spinal cord injured patients when stimulating the sacral roots (Rijkhoff et al., 1998) by inducing hyperpolarization of larger fibers at the anodic site, but successful long-term implantation studies have not been reported. Compared to our stimulation approach, anodal block requires longer monophasic pulse widths, which may lead to nerve damage due to charge imbalance if applied for long periods, but may reduce effects caused by the onset of the kilohertz frequency stimulation, and require either a bipolar or tripolar electrode instead of 2 pairs of electrodes. However, it remains unclear how well anodal block can be used to achieve selective activation of parasympathetic fibers over Aδ fibers and C fibers.

Although brief onset EUS EMG responses were observed during our blocking experiments, this onset EUS activity was temporally isolated from bladder contractions by initiating the KHFS 10 s prior to the low frequency stimulation. Some remnant EUS activities were still observed during the low frequency stimulation, possibly due to partial blocking effects. Additionally, the intact afferent pathway of the other pelvic nerve could also contribute to evoking EUS activity during bladder contraction, as KHFS of the pelvic nerve was unilateral. In rats, unlike cats and humans, the EUS exhibits oscillatory burst contractions to aid the flow of urine through the urethra and out via the urethral meatus. We have also observed this type of oscillatory EUS bursts during bladder filling-evoked or spontaneous voiding (see Supplementary Figure 2A), but it was not always present (see Figure 4C). This could be due to the effect of ketamine and xylazine suppressing lumbar (L3-4) spine-level circuits responsible for generating the EUS oscillatory bursts (Chang et al., 2007). While our combined low frequency stimulation and KHFS did not recapitulate EUS bursts during voiding, it is possible to artificially induce beneficial EUS burst activity via timed electrical stimulation of the pudendal nerve in rats and cats (Langdale and Grill, 2016), or more central sections of the pelvic nerve lateral to the KHFS blocking site.

As the blocking efficacy showed significant correlation with the ratio of KHFS blocking amplitude to 10 Hz stimulation amplitude (see Figure 7), this new strategy of stimulating pelvic nerves can theoretically be improved if higher blocking currents could be used to achieve complete block. Our nerve transection experiments indicated that 10 Hz stimulation current up to 400 μA can be used to drive detrusor contractions without dyssynergia in the total absence of evoked nerve signals traveling to the spinal cord (see Figure 4). However, in our study, we observed that high KHFS current amplitudes led to sustained excitatory EUS responses instead of inhibition (see Figure 5). As the KHFS nerve block was performed using bipolar electrodes in our study, higher current amplitudes could have generated sufficient potential to excite fibers outside the leads (Rattay, 1986; Brocker and Grill, 2013), giving rise to the impression that axonal fibers within the bipolar leads were excited. In order to ameliorate this issue in the future, the use of tripolar stimulation configuration could be used to decrease the excitation of additional nerve fibers, and increase the current range for the KHFS block.

It remains unclear what caused the progressive “carry-over” effect or partial reversibility of the KHFS block of the pelvic nerve (see Figure 6G and Supplementary Figure 3). Unfortunately, the length of this “carry over” effect could not be systematically determined in our study due to the constraints of experimental time. Based on the typical experimental length, the carry-over effects were estimated to vary from minutes to hours, and could depend on the duration of the kilohertz frequency stimulation. We did not observe any discoloration of the nerve or electrodes during our experiments that could indicate formation of toxic electrochemical by-products (Brummer and Turner, 1975) due to excessive high currents or charge imbalance. However, the possibility that portions of the nerve were damaged as a result of the kilohertz frequency stimulation cannot be excluded at this point. Previous studies using kilohertz frequency nerve block on other nerve targets had also reported blocking effects that lasted after the stimulation had ceased (Waataja et al., 2011; Yang et al., 2017). The exact mechanisms underlying reversible nerve conduction blocking caused by KHFS remains unclear, although constant activation of potassium channels has been proposed (Zhang et al., 2006a). Additional experiments or modeling will be required to examine if the state of the ion channels, or the intracellular ion concentrations, were disrupted far longer than the stimulation period to account for such carry-over effects. A potential caveat of the KHFS of the pelvic nerve is that the carry-over effects could diminish sensations of bladder fullness that could be used to decide when to voluntarily initiate pelvic nerve stimulation. Nonetheless, it is worth noting that this carry-over effect could also imply that the KHFS stimulation may be temporarily omitted from the combined low and high frequency stimulation paradigm to conserve power in stimulation devices in cases where significant blocking effect lasted much longer than the stimulation period.

Despite the increased complexity of the pelvic plexus in humans, there has been progress in the surgical localization of preganglionic pelvic nerves in humans using laparoscopy, in which intraoperative electrical stimulation of gently dissected nerves arising out of sacral roots elicited isolated increases in intravesical pressure (Possover et al., 2007). Nonetheless, more studies are needed beyond studying the pelvic nerves from cadavers (Yamaguchi et al., 2011; Spradling et al., 2015) to performing intra-operative mapping to identify suitable nerve candidates that can cause detrusor contraction, and are at least visible with endoscopy. Smaller nerves, such as the smaller postganglionic branches near the bladder, that are not visible with surgical magnification will indeed not be suitable targets as their “invisibility” increases the risk of injury that can lead to bladder dysfunctions as shown in some patients undergoing pelvic surgeries (Ripperda et al., 2017). Targeted electrical stimulation of human pelvic splanchnic nerves located near the bladder could also be complicated by the mixing of parasympathetic and sympathetic fibers in most branches, although fiber-specific nerves were also observed (Hinata et al., 2014). A plausible bridging step toward understanding clinical feasibility may be to first conduct experiments using bigger mammalian models with more similar anatomical features (Langley and Anderson, 1895) as technical questions such as how to target the pelvic nerves before they merge into the complex pelvic plexus, and how many nerve fibers need to be stimulated in order to achieve clinical benefit still remain unanswered. Availability of new electrodes capable of interfacing with small nerves (Lee et al., 2017; Lissandrello et al., 2017) will also push forward the field of bioelectronic medicine involving the autonomic nervous system. Our results indicate that using electrodes that maintained some extra-neural contact with the mixed pelvic nerves (see Figure 1B), and using the appropriate stimulation in the rat model, was sufficient to achieve selective activation of the efferent pathway. This finding could provide guidance for designing future implantable electrode interfaces (Navarro et al., 2005) targeting the mixed pelvic nerve branches with parasympathetic and sensory fibers in larger animals or humans in the future.

Future work will aim to examine the efficacy of this novel neurostimulation strategy for restoring voluntary bladder voiding in animal models with a loss of supraspinal control of micturition. Examples include spinal cord injuries, or models with peripheral nerve injury or neuropathy that require augmented stimulation of bladder efferent inputs, or animals with underactive bladder, which currently lack effective treatments other than catheterization (Tyagi et al., 2014). A more cogent demonstration of the applicability of this technique will also involve stimulation in the awake state to remove any confounds related to the suppression of spinal circuits by anesthesia. Chronic studies would also be important to elucidate how combined low and high frequency stimulation of pelvic nerve affects neural plasticity known to occur at the spinal level circuits after injury (de Groat and Yoshimura, 2006, 2012).

## Author contributions

WP: designed and performed experiments, analyzed data, and wrote the manuscript; RM, XT, SC, and AR: performed experiments and reviewed the manuscript; NT and S-CY: conceived the experiments, provided guidance, and reviewed the manuscript.

### Conflict of interest statement

The authors declare that the research was conducted in the absence of any commercial or financial relationships that could be construed as a potential conflict of interest.
