# Supplementary material for: Novel Neurostimulation of Autonomic Pelvic Nerves Overcomes Bladder-Sphincter Dyssynergia
Source: Front Neurosci. 2018 Mar 21;12:186. doi: 10.3389/fnins.2018.00186 (PMC5871706; doi:10.3389/fnins.2018.00186)
Supplement: Supplementary file 1 [file Presentation1.pdf]

Supplementary Material

Novel neurostimulation of autonomic pelvic nerves overcomes bladder-sphincter dyssynergia

Wendy Yen Xian Peh[1], Roshini Mogan[1], Xin Yuan Thow[1], Soo Min Chua[1], Astrid Rusly[1], Nitish V. Thakor[1,2,3,4*], Shih-Cheng Yen[1,3*]

[1]Singapore Institute for Neurotechnology, National University of Singapore, Singapore

[2]Department of Biomedical Engineering, National University of Singapore, Singapore

[3]Department of Electrical and Computer Engineering, National University of Singapore, Singapore

[4]Biomedical Engineering, School of Medicine, John Hopkins University, Baltimore, MD, USA

*** Correspondence:**
Dr. Shih-Cheng Yen
shihcheng@nus.edu.sg

Dr. Nitish V. Thakor
sinapsedirector@gmail.com

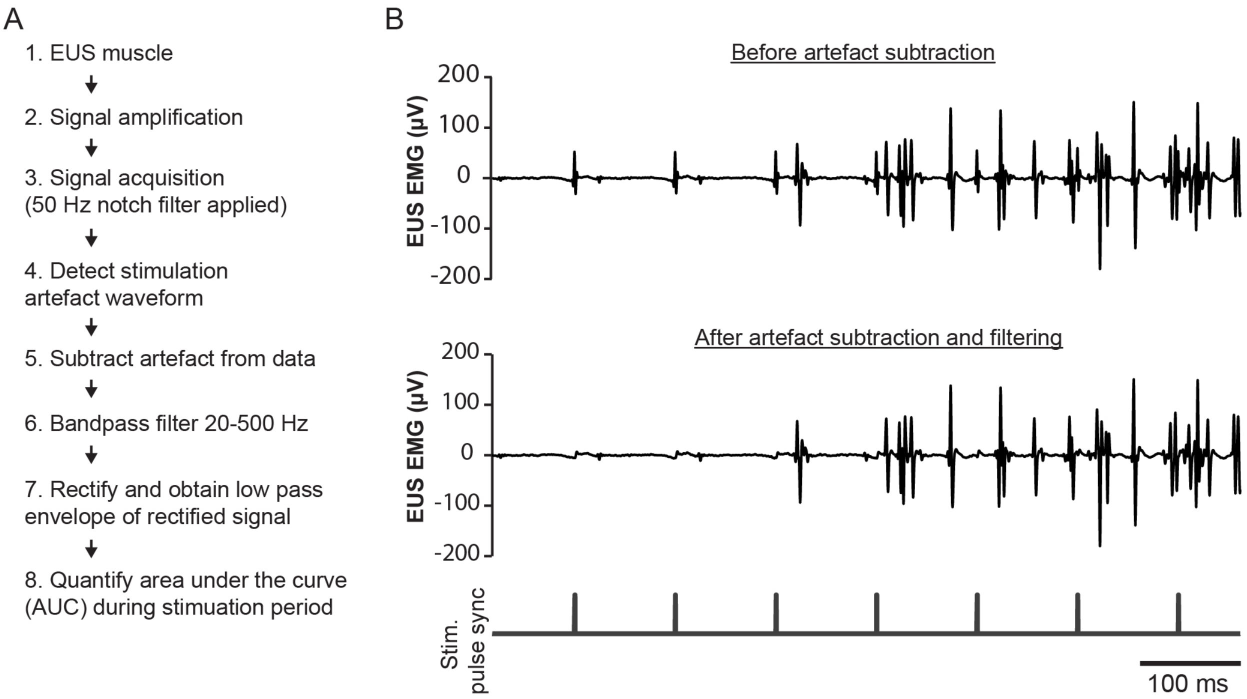

Supplementary Figure 1. EUS EMG signal processing, and reduction of stimulation artefact. **(A)** Sequence of signal processing and quantification of EMG data collected for experiments with 10 Hz, 5 seconds of stimulation, with or without the high frequency block. **(B)** Example of EMG recording before, and after artefact subtraction and filtering. Stimulation pulse onsets are indicated below the EMG traces.

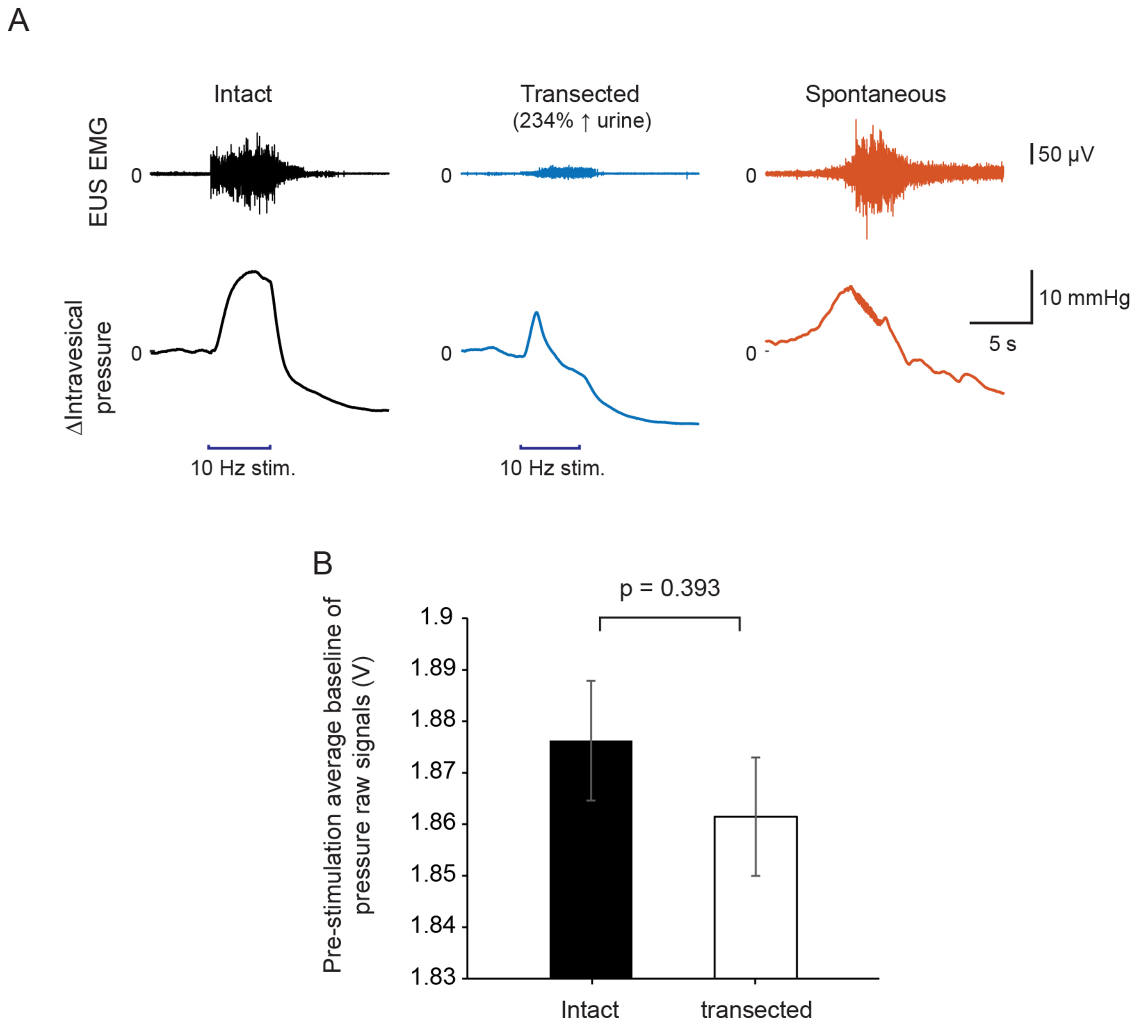

Supplementary Figure 2. **(A)** Example of EUS EMG recordings and bladder responses to either stimulation of intact pelvic nerve (black traces), stimulation of the proximally transected pelvic nerve (blue traces), or spontaneous voiding (orange traces). Percentage increases in urine volume when stimulating transected nerves compared to intact nerves are indicated in parenthesis above the blue trace. **(B)** The baseline pressure signals were not significantly different between trials with intact and transected nerves ($p = 0.393$, paired t-tests, $N = 5$).

Supplementary Table 1: Electrical stimulation and blocking parameters used for the data shown in Fig. 8, and the corresponding decreases in the evoked EMG.

		Low frequency stimulation			Kilohertz frequency block			
Rat #	Case #	Current amplitude (μA)	Phase width (μs)	Duration (s)	Current amplitude (μA)	Phase width (μs)	Duration (s)	% reduction in evoked EMG
1	1	50	150	5	400	25	16	76.02
1	2	75	150	5	400	25	16	79.07
1	3	75	300	5	400	25	16	57.90
2	4	300	150	5	700	25	16	48.19
3	5	50	300	5	600	25	16	37.00
4	6	75	150	5	300	25	16	32.87

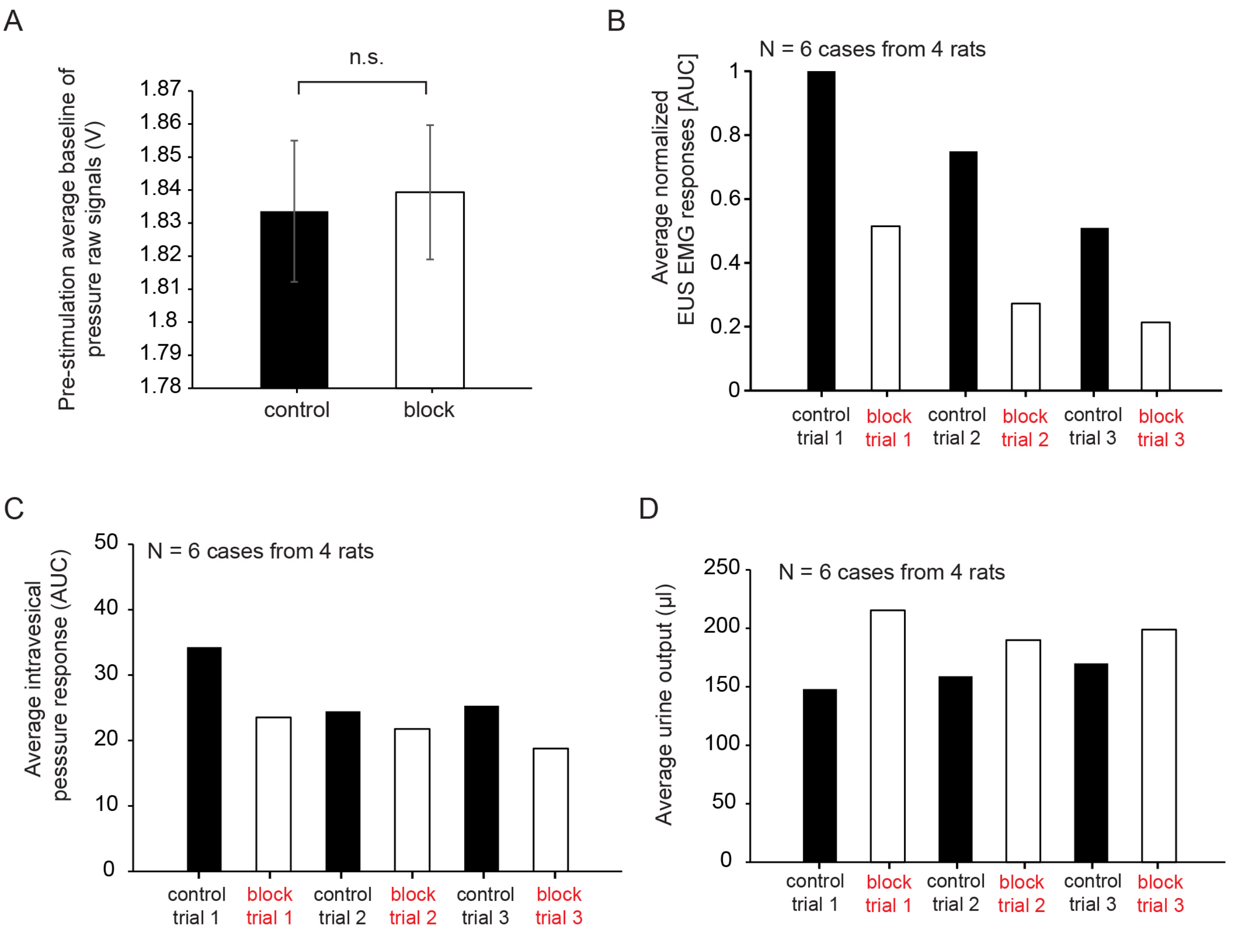

Supplementary Figure 3. (A) The baseline pressure signals were not significantly different between the interleaved control and block trials (p = 0.257, paired t-tests, n = 6 cases in 4 rats). **(B)** Average normalized EUS EMG responses (area under curve during stimulation) obtained from 6 consecutive control and blocking trials from 4 rats. Evoked EMG response was normalized to the maximum value obtained within the 6 trials (3 control trials + 3 block trials) for each case. **(C)** Average intravesical pressure changes (area under curve during stimulation) obtained from 6 consecutive control and blocking trials from 4 rats. **(D)** Average urine output obtained from 6 consecutive control and blocking trials from 4 rats.
